# Reduction of Hospitalization and Mortality by Echocardiography-Guided Treatment in Advanced Heart Failure

**DOI:** 10.3390/jcdd9030074

**Published:** 2022-03-03

**Authors:** Hamayak Sisakian, Syuzanna Shahnazaryan, Sergey Pepoyan, Armine Minasyan, Gor Martirosyan, Mariam Hovhannisyan, Ashkhen Maghaqelyan, Sona Melik-Stepanyan, Armine Chopikyan, Yury Lopatin

**Affiliations:** 1 Clinic of General and Invasive Cardiology, University Hospital #1, Yerevan State Medical University, Yerevan 0025, Armenia; syuzanna.shahnazaryan@gmail.com (S.S.); spepoyan@gmail.com (S.P.); g.martirosyan29@gmail.com (G.M.); mariamhovhannisyan.r@gmail.com (M.H.); doc.mag88@mail.ru (A.M.); 2 Surb Grigor Lusavorich Medical Centre, Yerevan State Medical University, Yerevan 0025, Armenia; armine_minasyan@yahoo.com (A.M.); sonarsh@mail.ru (S.M.-S.); 3 Public Health and Healthcare Organization Department, Yerevan State Medical University, Yerevan 0025, Armenia; arminchopikyan@gmail.com; 4 Volgograd Regional Cardiology Centre, Volgograd State Medical University, 400131 Volgograd, Russia; prof_lopatin@mail.ru

**Keywords:** advanced heart failure, outpatient monitoring, Tissue Doppler echocardiography, left ventricular filling pressure, mortality, rehospitalizations

## Abstract

In advanced heart failure (AHF) clinical evaluation fails to detect subclinical HF deterioration in outpatient settings. The aim of the study was to determine whether the strategy of intensive outpatient echocardiographic monitoring, followed by treatment modification, reduces mortality and re-hospitalizations at 12 months. Methods: 214 patients with ejection fraction < 30% and >1 hospitalization during the last year underwent clinical evaluation and echocardiography at discharge and were divided into intensive (IMG; N = 143) or standard monitoring group (SMG; N = 71). In IMG, volemic status and left ventricular filling pressure were assessed 14, 30, 90, 180 and 365 days after discharge. HF treatment, particularly diuretic therapy, was temporarily intensified when HF deterioration signs and E/e’ > 15 were detected. In SMG, standard outpatient monitoring without obligatory echocardiography at outpatient visits was performed. Results: We observed lower hospitalization (absolute risk reduction [ARR]-0.343, CI-95%: 0.287–0.434, *p* < 0.05; number needed to treat [NNT]-2.91) and mortality (ARR-0.159, CI 95%: 0.127–0.224, *p* < 0.05; NNT-6.29) in IMG at 12 months. One-year survival was 88.8% in IMG and 71.8% in SMG (*p* < 0.05). Conclusion: In AHF, outpatient monitoring of volemic status and intracardiac filling pressures to individualize treatment may potentially reduce hospitalizations and mortality at 12 months follow-up. Echocardiography-guided outpatient therapy is feasible and clinically beneficial, providing evidence for the larger application of this approach.

## 1. Introduction

Advanced heart failure (AHF) is a clinical syndrome characterized by persistent heart failure (HF) symptoms and progressive left ventricular (LV) dysfunction, despite guideline-based medical therapy [1,2,3,4]. Patients with advanced progressive HF have frequent hospital readmissions due to pulmonary or systemic congestion, high mortality and poor quality of life [2,3]. In severe HF, recurrent hospitalizations increase the risk of adverse events, therefore, the concept of preventing hospital readmissions in this patient population has gained considerable importance over the last few years [5].

Worsening of HF signs and symptoms due to increasing fluid retention and congestion precede hospitalizations for acute HF decompensation. In ambulatory patients, progressive dyspnoea, weight gain, peripheral oedema and crackles on lung auscultation are suggestive of HF deterioration and are important predictors of the upcoming HF decompensation and hospitalization [6]. However, clinical evaluation and lung auscultation may fail to detect subclinical HF decompensation [7,8,9]. On the other hand, intracardiac hemodynamic changes and an increase in LV filling pressure precede the manifestation of HF symptoms and can be detected by echocardiography [10,11,12]. Increased ventricular filling pressure results in high atrial pressure with subclinical HF deterioration [13,14]. Therefore, the possibility to assess filling pressure in ambulatory patients with AHF provides accurate diagnosis of subclinical decompensation, expanding the already established role of echocardiography in heart failure. Early identification of the vulnerable period is important for the timely adjustment of a therapeutic regimen to prevent HF decompensation-related rehospitalizations in AHF patients.

The purpose of this study was to support our hypothesis that the strategy of outpatient echocardiography-guided treatment improves the efficacy of care and outcomes of patients with AHF at 12 months follow-up.

## 2. Materials and Methods

### 2.1. Study Design and Population

The prospective study was conducted among the patients corresponding to AHF definition of the European Society of Cardiology (ESC) [2] between January 2017 and February 2018. Eligibility criteria for intensive outpatient echo-guided monitoring included: (1) age between 18 and 90 years, (2) symptomatic patients with NYHA III–IV functional class, despite the optimal guideline-based therapy, (3) more than 1 hospitalization for HF decompensation within the past 12 months, (4) LV ejection fraction (EF) < 30% documented by transthoracic echocardiography, (5) left atrial (LA) volume > 35 mL/m^2^, and (6) E/e’ > 15 determined by Tissue Doppler (TD) before hospital discharge. Exclusion criteria were: (1) acute myocardial infarction or unstable angina in the past 3 months, (2) severe valvular disease, (3) inability to follow the study protocol, and (4) dialysis.

Before being discharged from hospital, patients were divided in two parallel groups in a ratio 2:1: intensive monitoring group (IMG) with mandatory outpatient echocardiography and standard monitoring group (SMG) with detailed physical examination only.

**Figure 1:**
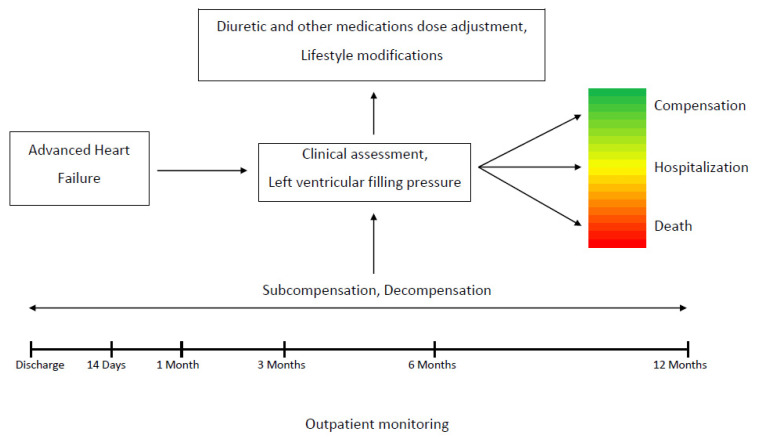
Workflow chart of the present study.

Outpatient evaluation and echocardiography with filling pressure assessment were provided by experienced HF specialists and trained echocardiography physicians according to ESC HFA and ASE guidelines [15,16]. A workflow chart of the present study is presented in Figure 1.

A total of 249 patients admitted with HF decompensation were recruited between January 2017 and February 2018 in two University affiliated hospitals in Yerevan, Armenia. The data obtained from 214 patients, who completed the 12 months follow-up period, were used for statistical analysis. The study was approved by the local Ethics Committee and complied with Declaration of Helsinki principles. Informed consent was obtained from all participants before enrolment.

### 2.2. Clinical Data and Study Protocol

Comorbidities and clinical data of each patient were obtained from patient examination and hospital medical records. Congestion was assessed by presence of crackles on lung auscultation and peripheral oedema. The glomerular filtration rate (GFR) was calculated based on the creatinine levels at discharge using the Cockcroft–Gault equation.

Patients, who met the requirements after the discharge were assigned to intensive or standard monitoring groups and were followed-up for 12 months.

Intensive monitoring included five consecutive hospital visits 14, 30, 90, 180 and 365 days after the discharge. At discharge and during the visits, patients underwent a physical examination, body mass assessment, heart rate, GFR, and electrocardiography (ECG). Echocardiographic evaluation of the LV filling pressure and LA volume were part of intensive monitoring strategy at each outpatient visit. Based on the clinical evaluation of volemic state and echocardiographic data, patients with signs of worsening congestion and high LV filling pressure (E/e’ > 15) were managed by intensification of diuretic treatment (administration of double doses of oral loop diuretics or additional intravenous low dose 20–40 mg furosemide). Additional modifications of HF therapy included switching from renin-angiotensin-aldosterone system (RAAS) inhibitor to sacubitril/valsartan, change of prescribed doses of beta-blockers, and mineralocorticoid receptor antagonists (MRA). Other evidence-based pharmacologic HF therapies were administered per regular protocol during the outpatient period. Patients were hospitalized if treatment modification was not effective in the prevention of HF decompensation.

In SMG, standard outpatient monitoring was performed with the same terms and frequency of hospital visits, but without obligatory echocardiographic study. Outpatient follow-up visits included only physical assessment of congestion and ECG. Decision of treatment modification was based on physical examination only.

### 2.3. Echocardiographic Evaluation

An experienced cardiologist with everyday practice in echocardiography conducted transthoracic follow-up echocardiographic examinations. The echocardiographist was blinded to the patients’ treatment group. Basic and advanced echocardiographic assessments were performed using a commercially available system (General Electronic [GE] Vivid E9; 2017). All dimension and volume measurements were performed in accordance with accepted guideline recommendations [16,17]. The main treatment targets in the intensive treatment group were E/e’ < 15 and resolution of clinical congestion for the prevention of rehospitalizations. After 5–7 days of short-term loop diuretic treatment intensification, patients returned to previous treatment doses if E/e’ ratio was <15 and signs of congestion were absent.

LA volume was measured from 2-chamber and 4-chamber apical views with the area-length biplane method. Early diastolic velocity of transmitral flow (E) was measured in apical 4-chamber view using the Doppler technique. Septal early diastolic mitral annular velocity (e’) was measured using TD. In patients with atrial fibrillation, E and e’ waves were measured in five consecutive cycles and their average value was used to calculate E/e’ [16].

Investigators were unblinded to group assignment. However, echocardiographic assessment was carried out blindly. Medical history, clinical examinations and treatment changes were recorded at baseline and at each visit during the 12-month follow-up.

### 2.4. Study Endpoints

All patients were followed-up for 12 months. The primary endpoints were hospitalization for HF decompensation and mortality from cardiac causes. HF hospitalizations were defined as hospital admissions for worsening signs and symptoms of HF with more than 24 h hospital stay. Cardiovascular death included death from acute myocardial infarction, HF, cardiovascular procedures, and sudden cardiac death.

### 2.5. Statistical Analysis

Statistical analysis was performed using the IBM_22.0.0 SPSS statistical package (IBM, Armonk, NY, USA). Continuous variables with normal distribution were expressed as mean, standard deviation (SD), standard error (SE) and categorical variables presented as numbers and percentages. All *p* values were from 2-tailed tests, and results were deemed statistically significant at *p*  < 0.05. For the evaluation of effectiveness of new approach (intensive ambulatory monitoring), absolute risk reduction (ARR) and number needed to treat (NNT) were used.

Survival assessment was estimated by the Kaplan–Meier method with adjustment for baseline differences in covariates and was compared using the long rank test.

Due to the non-randomized nature of the study, the propensity score (PS) was performed in order to minimize selection bias between groups. However, both echocardiographic examination and study endpoint assessments were carried out blindly. PS estimates the likelihood of receiving standard or intensive monitoring based on the clinical characteristics of each patient. The covariates included in the logistic regression analysis for PS calculation were age, gender, NYHA class, LA volume index, E/e’ ratio, beta-blockers, digoxin, and angiotensin receptor antagonist/neprilysin inhibitor at baseline, etc.

## 3. Results

### 3.1. Baseline Characteristics

Patients baseline characteristics are presented in Table 1.

The mean age of all study subjects was 65.9 ± 10 years, 78.5% of the patients were men. A total of 100 patients in IMG (70%) and 64 in SMG (90%) had a history of coronary artery disease.

Patients in IMG had more severe clinical risk markers compared to SMG. They were also likely to have more severe disease (42% of patients in IMG had NYHA class IV vs. 31% of patients in SMG).

Patients in IMG had higher mean E/e’ ratio (24.1 ± 6.9), higher mean LA volume index (51.7 ± 19.1), and lower mean EF (20.1 ± 5.2) at baseline. Baseline medical treatment of the patients is presented in Table 2.

In the IMG group 62 (46.6%), patients underwent an increase in doses and switch to i.v. furosemide at 3 months, 54 (42.2%) at 6 months and 35 (27.8%) at 12 months, whereas in SMG only 4 (7.0%) patients received i.v. furosemide treatment at 3 months and 3 (5.9%) patients at 6 months (Table 3). In SMG, no patients were prescribed i.v. furosemide at 12 months. Despite more frequent switch to i.v. furosemide in IMG, the mean i.v. furosemide doses in both groups at follow-up visits did not differ significantly and were even higher at some time periods in SMG (Table 3).

### 3.2. Outcomes

We observed a total of 93 hospitalizations for HF decompensation over the 12 months follow-up, including 43 (30%) in the IMG, and 50 (70%) in the SMG. Eight (6%) patients in IMG and 14 (19.7%) patients SMG had more than one hospitalization for HF decompensation within the study period.

Hospitalizations for other reasons, such as stroke, cancer and implantable cardioverter-defibrillator (ICD) implantation occurred in three patients in SMG.

**Figure 2:**
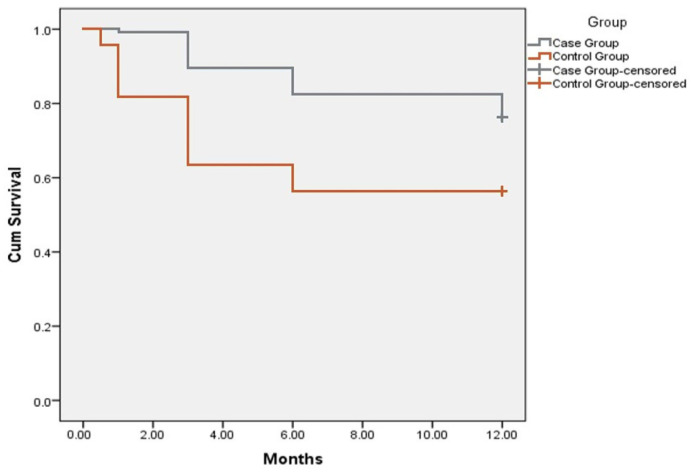
Kaplan–Meier curve showing hospitalization rates in intensive monitoring group compared to standard monitoring (control) group during 12 months follow-up period. Y axis represents Cum Survival, X axis—follow-up period represented by months.

We observed a statistically significant (*p* < 0.05) reduction in hospitalization rates for HF decompensation in IMG. Compared to the SMG, significantly lower hospitalization rates were observed in IMG at 1, 3 and 6 months after discharge (Figure 2).

A total of 20 (28.2%) deaths were observed in SMG, including two cases of deaths from non-cardiac causes. In IMG, 16 deaths (11.2%) occurred during the follow-up, among them 13 individuals died from cardiovascular causes and three patients from cancer. The causes of cardiovascular deaths were acute myocardial infarction in three patients, sudden cardiac death in two, and HF decompensation in eight patients. Compared to SMG, we observed a significant decrease in mortality rates in IMG at 3, 6 and 12 months of follow-up with a significantly lower mortality rates at 12 months after the discharge (*p* < 0.05).

**Figure 3:**
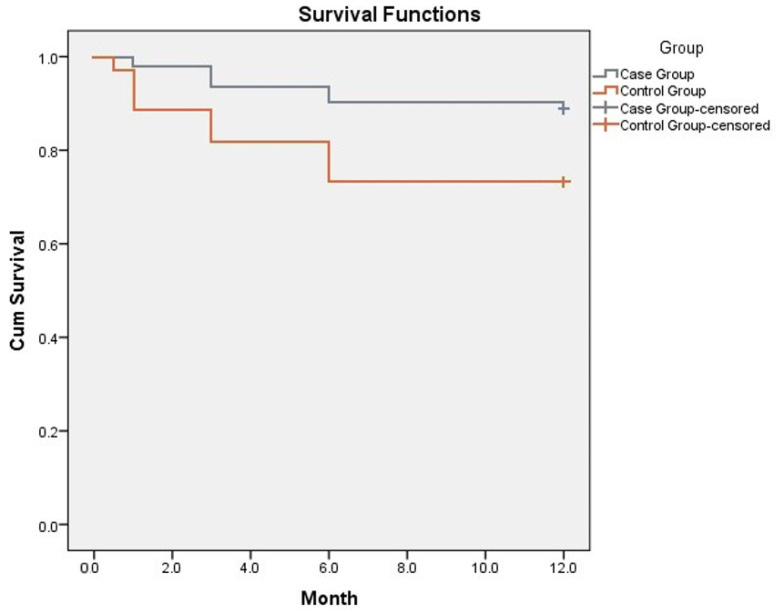
Kaplan–Meier curve showing survival rates in intensive and standard monitoring groups. Y axis represents Cum Survival, X axis—follow-up period represented by months.

Kaplan–Meier curve for 1-year survival showed an improved survival in IMG. Mean survival in the intensive monitoring group was 11.2 ± 0.22 months (CI 10.75–11.61) and in the standard monitoring group 9.61 ± 0.49 months (CI 10.75–11.61) (Figure 3). The data suggested that 1-year survival was 88.8% in the IMG and 71.8% in the control group (*p* < 0.05).

The absolute risk reduction for rehospitalizations was 0.36, and the number needed to treat (NNT) was 2.78, indicating that a total of 2.78 patients would need intensive monitoring (rather than standard) for one additional patient to avoid rehospitalization over a 12-month period. When corrected with PS, absolute risk reduction remained significant (ARR = 0.343, CI 95%: 0.287–0.434, *p* < 0.05). PS was not significantly associated with risk reduction (*p* > 0.05). After correction NNT was 2.91.

The absolute risk reduction for survival was estimated at 0.17, and the NNT was 5.88, indicating that a total of 5.88 patients would need an intensive monitoring (rather than standard) for one additional patient to survive over a 12-month period. When corrected with PS, absolute risk reduction remained significant (ARR = 0.159, CI 95%: 0.127–0.224, *p* < 0.05). PS was not significantly associated with risk reduction (*p* > 0.05). After correction, NNT was 6.29.

## 4. Discussion

Patients with AHF remain at high risk of death and rehospitalizations despite advances in HF therapy. During the last decade, it has become evident that ambulatory monitoring of patients with HF leads to fewer decompensations. Furthermore, a personalized approach remains an important step towards the achievement of a better outcome [18]. The implementation of focused echocardiography in such settings is supported both by previous and current ESC guidelines on Heart Failure and ASE guidelines [15,16,19].

Frequently, haemodynamic deterioration precedes clinical congestion by days or weeks [20]. Therefore, clinical congestion may be seen as the “tip of an iceberg” of the haemodynamic compromise. Moreover, prolonged elevated filling pressure predisposes to organ injury and hypoperfusion, neurohormonal and proinflammatory response. Multiorgan dysfunction, particularly renal and hepatic failure, contributes to poor survival in this patient group [21]. Determination and quantification of haemodynamic congestion are crucial steps in the examination of HF patients. Thus, the failure to recognize subclinical elevation of pulmonary capillary wedge pressure (PCWP) may negatively affect the prognosis of HF patients [22]. An increase in LV filling pressure preceding the onset and aggravation of HF symptoms may be easily detected by echocardiography in patients with subclinical HF decompensation as an early predictor of HF deterioration [10]. Therefore, recognition of an increase in LV filling pressure at outpatient settings may enable early identification of AHF patients in the “vulnerable phase” of subclinical decompensation [23]. This offers a window for a timely escalation of diuretic therapy for the prevention of rehospitalizations.

Echocardiography is a safe, accessible and low-cost examination technique, which can be successfully used for the detection of subclinical HF decompensation. However, echocardiographic monitoring continues to have a limited application in outpatient settings.

Comprehensive echocardiography with TD imaging overcomes the limitations of physical examination and clinical picture-based strategies for the prediction of HF rehospitalizations. Moreover, the latter has been reported to have low sensitivity and efficacy in the early identification of upcoming hospital readmissions [8,9].

Over the last few decades, multiple prospective multicentre studies have investigated remote monitoring approaches based on the assessment of body weight, symptoms, blood pressure, heart rate, and capillary oxygen saturation. However, analyses of these studies show no consistent benefit of non-hemodynamic monitoring in HF patients [24,25,26,27,28]. In AHF patients, cardiac filling pressures rise several weeks before deterioration and hospitalization. Meanwhile, symptoms of clinical congestion occur usually shortly before the hospitalization [29]. This has led to the development of several invasive monitoring devices during the last years.

Invasive guidance of decongestive therapy guided by pulmonary artery pressure was associated with a significant reduction in hospitalizations in outpatient chronic HF patients in the CHAMPION trial [30] However, invasive monitoring has several limitations with regard to availability and safety. Moreover, in the ESCAPE trial, treatment of patients with acute HF, guided by pulmonary artery catheter, was non-beneficial compared with conventional therapy [31]. The US Post Approval Study (PAS) showed a 58% reduction in HF-related hospitalizations in the first year after CardioMEMS implantation compared with 1 year before implantation. Furthermore, a reduction in HF hospitalizations, mortality and all-cause mortality was observed after CardioMEMS implantation. However, patients included in the PAS study were their own historical controls and there has been no randomized comparison to standard care without pulmonary artery monitoring [32,33,34].

To date, there are several studies on cardiothoracic ultrasound monitoring aimed to provide decongestion in patients with acute and chronic HF (Table 4). In these studies, different patient groups were assessed by several measurements. In most of the studies, patients in the cardiothoracic-guided treatment arm seemed to be more effectively decongested compared to patients on standard treatment. However, E/e’ parameter and LA re-modelling assessments provide a more integrated approach among different cardiac ultra-sound measurements and are applied in routine clinical use. Thus, our study validated echo-guided parameters and demonstrated the reliable efficacy of echocardiography-guided treatment in ambulatory specific patient groups.

The simplicity of echocardiographic application and measurements makes it potentially useful for larger groups of HF patients at relatively lower risk, where invasive telemonitoring may have more impact in sicker patients.

One of the main objectives of our study was to prevent frequent hospitalizations in patients with AHF. The optimization of treatment using non-invasive easy monitoring should be implemented for such patients at outpatient periods.

In our study of patients with advanced HF, NYHA III–IV and LVEF < 30%, intensive outpatient monitoring of LV filling pressures with need-based adjustment of HF treatment was associated with improved short and long-term survival and lower risk of rehospitalizations. The substantial reduction in both hospitalization and mortality were observed predominantly at a 3–6 months period. The data analysis suggested an improvement in 1-year survival with an absolute risk reduction of hospitalizations and mortality in IMG.

In our opinion, the main diagnostic tool of the observed beneficial effects is the assessment of LV filling pressures as early predictive parameters of HF decompensation, which precedes clinical deterioration. Changes in intracardiac hemodynamics and LV filling pressure elevation precede the development of symptoms and signs of congestion, and, finally, lead to HF hospitalization [10]. In our study, LA volume and E/e’ measurements allowed early identification of LV filling elevation and intracardiac hemodynamic worsening and predicted HF decompensation in subclinical patients. Thus, in terminally ill patients, intensive echocardiographic monitoring with repeated measurement of LA volume and E/e’ assessment by TD echocardiography may predict and prevent HF deterioration and, therefore, may lead to the reduction of hospitalizations and improvement of the prognosis.

In IMG, diuretic treatment intensification was guided by repeated blind outpatient echocardiographic assessments of LV filling pressures. Specifically, loop diuretic dose up-titration and/or addition of intravenous furosemide was provided in accordance with E/e’ ratio increase and volemic status. On the other hand, in SMG, the decision of changing diuretic dose was based on the clinical picture and physical examination alone. Thus, repeated echocardiographic measurements of LV filling pressures in patients with AHF may warranty a targeted and timely intensification of diuretic therapy. This fact can explain the difference in outcomes in two arms, particularly a statistically significant reduction of hospitalization and mortality rates in IMG.

Our findings are in line with our initial hypothesis, that echocardiography-guided therapy would improve outcomes and reinforce the importance of echocardiography-detected subclinical congestion in the management of AHF patients. TD examination is a quick and easy to perform technique which is available in most cardiology departments. The results of this study highlight that currently, many patients may be receiving inadequate treatment and may probably be undertreated. Therefore, based on the results of the study, HF specialists are encouraged to use LV filling pressure measurements more frequently for the detection of subclinical HF deterioration and to enable more effective and timely treatment of these patients.

### Study Limitations

As all observational studies our study carries the possibility for bias, leading to an over or underestimation of the intensive monitoring approach. The unblinded design of the study may represent a potential limitation. Although two groups of study populations were homogeneous regarding the main baseline characteristics, we provided a propensity score calculation to minimize selection bias.

Participating physicians were free to choose an intensive versus standard outpatient monitoring approach. The baseline differences in several clinical parameters, such as atrial fibrillation, NYHA class IV, E/e’ ratio, and treatment with MRA suggest, that physicians were more likely to provide intensive monitoring and follow-up to high-risk patients with more severe disease. Despite the higher baseline risk of patients in IMG, mortality and hospitalization rates were lower in this group compared to SMG. Besides the expected differences in NYHA class and E/e’ ratio, we observed significantly more cases of chronic kidney disease in IMG. Another limitation of study was exclusion of severe valvular heart disease, which may limit generalization of our results to such patients.

## 5. Conclusions

Hemodynamic monitoring by echocardiography is a widely available and cost-effective method for the outpatient assessment of patients with AHF. It can be implemented at different stages of care for the identification of vulnerable, high-risk patients, allowing optimal and timely adjustment of therapeutic regimens for the prevention of rehospitalizations. TD examination of LV filling pressures provides risk assessment for patients with subclinical decompensation prior to the clinically apparent HF decompensation. Frequent echocardiographic monitoring of filling pressures in AHF patients may lead to a considerable reduction in recurrent hospitalizations and mortality at a 12-month period.

## Figures and Tables

**Table 1:** Patients baseline characteristics in intensive and standard monitoring groups.

Variable	Intensive Monitoring Group (*n* = 143)	Standard Monitoring Group (*n* = 71)	*p* Value
Age (years)	66.6 ± 10.1	64.6 ± 10.1	0.186
Women (*n*, %)	30 (21)	16 (23)	0.794
Body mass (kg)	84.6 ± 14.2	80.5 ± 13.3	0.043
Coronary artery disease (*n*, %)	100 (70)	64 (90)	0.001
Diabetes mellitus (*n*, %)	37 (26)	18 (25)	0.934
CKD (*n*, %)	51 (36)	16 (23)	0.051
Heart rate (beats per minute)	84.8 ± 15.0	79.2 ± 17.4	0.016
Sinus rhythm (*n*, %)	93 (65)	54 (76)	0.102
Atrial fibrillation (*n*, %)	48 (34)	17 (24)	0.149
Pacemaker (*n*, %)	2 (1)	0 (0)	0.317
ICD (*n*, %)	3 (2)	2 (3)	0.743
CRT (*n*, %)	4 (3)	0 (0)	0.155
Systolic blood pressure (mmHg)	117.2 ± 17.9	119.7 ± 17.4	0.326
Diastolic blood pressure (mmHg)	71.2 ± 10.5	75.3 ± 11.9	<0.001
Creatinine (mmol/L)	103.9 ± 38.3	130 ± 39.4	<0.001
Potassium (mmol/L)	4.6 ± 0.5	4.2 ± 0.6	0.027
NYHA class			
III	83 (58%)	49 (69%)	0.120
IV	60 (42%)	22 (31%)	0.120
Echocardiographic parameters			
LV ejection fraction (%)	20.1 ± 5.2	22.5 ± 4.0	0.001
LA volume index (mL/m^2^)	51.7 ± 19.1	46 ± 10.4	0.020
E/e’ ratio	24.1 ± 6.9	15.8 ± 2.2	<0.001

The data are expressed as mean ± SD (standard deviation). CKD—chronic kidney disease; ICD—implantable cardioverter-defibrillator; CRT—cardiac resynchronization therapy; NYHA—New-York Heart Association classification; LV—left ventricle; LA—left atrium.

**Table 2:** Medical treatment characteristics at baseline.

Medical Treatment	Intensive Monitoring Group (*n* = 143)	Standard Monitoring Group (*n* = 71)	*p* Value
Beta-blocker (*n*, %)	131 (91.6)	62 (87.3)	0.321
ACEi/ARB (*n*, %)	123 (86)	63 (88.7)	0.014
MRA (*n*, %)	136 (95.1)	62 (87.3)	0.042
Furosemide, oral (*n*, %)	138 (96.5)	66 (93)	0.247
Digoxin (*n*, %)	36 (25.2)	12 (16.9)	0.172
ARNI (*n*, %)	16 (11.2)	1 (1.4)	0.013
Inotropes (in-hospital) (*n*, %)	81 (57)	24 (33.4)	0.002
Vasodilators (*n*, %)	14 (9.7)	32 (45)	<0.0001

ACEi—Angiotensin-converting enzyme inhibitor; ARB—Angiotensin receptor blocker; MRA—Mineralocorticoid receptor antagonist; ARNI—Angiotensin receptor antagonist/neprilysin inhibitor.

**Table 3:** Diuretic doses at baseline and follow-up visit.

**Furosemide, Oral**		**at Discharge**	**3 Months**	**6 Months**	**12 Months**
IMG	Study group (*n*)	142	133	128	126
	Patient number (*n*, %)	141 (99.3%)	126 (94.7%)	123 (96.1%)	122 (96.8%)
	Mean dose (mg)	44.96 ± 16.76	59.52 ± 32.64	63.74 ± 35.61	65.74 ± 39.29
SMG	Control Group (*n*)	71	57	51	51
	Patient number (*n*, %)	66 (93%)	55 (96.5%)	36 (70.6%)	13 (25.5%)
	Mean dose (mg)	64.55 ± 23.48	58.91 ± 30.65	64.44 ± 34.84	61.54 ± 35.08
**Furosemide, i.v.**		**at discharge**	**3 months**	**6 months**	**12 months**
IMG	Patient number (*n*, %)	28 (19.7%)	62 (46.6%)	54 (42.2%)	35 (27.8%)
	Mean dose (mg)	25.71 ± 10.69	36.45 ± 21.89	43.70 ± 25.50	62.29 ± 36.23
SMG	Patient number (*n*, %)	25 (35.2%)	4 (7.0%)	3 (5.9%)	0
	Mean dose (mg)	64.8 ± 20.23	65.00 ± 25.17	86.67 ± 30.55	0
**Torasemide, Oral**		**at discharge**	**3 months**	**6 months**	**12 months**
IMG	Patient number (*n*, %)	61 (43.0%)	81 (60.9%)	83 (64.8%)	82 (65.1%)
	Mean dose (mg)	8.28 ± 3.28	10.43 ± 4.89	10.18 ± 4.58	10.06 ± 4.87
SMG	Patient number (*n*, %)	2 (2.8%)	3 (5.3%)	2 (3.9%)	2 (3.9%)
	Mean dose (mg)	10	10	10	10

The data are expressed as mean ± SD (standard deviation). IMG—intensive monitoring group; SMG—standard monitoring group.

**Table 4:** Review of studies on the role of cardiothoracic monitoring on prognosis in acute and chronic heart failure patients.

Author (Year)	Study Type	Number of Patients	Patient Characteristics	Methodology	Outcomes	Limitations and Pitfalls
Ohman J., Harjola V-P., 2018 [35]	Pilot, prospective	20	Acute HF, E/e’ > 15, pulmonary congestion	E/e’, IVC index, LUS	Decrease of all-cause death and acute HF rehospitalisation. Better decongestion of patients	Small pilot study with unequal population of patients in two groups
Rivas-Lasarte M., Alvarez-Garcia J., 2019 (LUS-HF) [36]	Randomized trial	123	HF, high NT-proBNP, pulmonary congestion	LUS	LUS-guided strategy reduced hospitalisations and mortality at 6-month follow-up	Treatment protocol was not exclusively based on LUS findings
Marini C., Fragasso G., 2020 [37]	Multicentre, randomized	244	Chronic HF outpatient	LUS	Mid-term reduction of hospitalisations with LUS-guided managements	Mid-term (90 days) follow-up
Pang P., Russel F., 2021 (BLUSHED-AHF) [38]	Multicentre, randomized	130	Acute HF	LUS	No benefit of LUS-guided strategy compared to usual care at 90 days. No benefit of B-lines < 15 after 6 h decongestion, however faster resolution of congestion after 48 h	No assessment of long-term rehospitalisation
Sisakian H., Shahnazaryan S., 2022	Prospective	214	Advanced chronic HF	E/e, LV filling pressure	Decrease of hospitalisations and mortality in echo-guided group by intensive monitoring at 12-month follow-up	Exclusion of patients with severe valvular disease. Preliminary un-blinded selection

HF—heart failure; IVC—inferior vena cava; NT-proBNP—N-terminal pro-B-type natriuretic peptide; LV—left ventricle; LUS—lung ultrasound.

## Data Availability

The data presented in this study are available on request from the corresponding author. The access numbers will be provided prior to the publication.
